# Finite Element Analysis of Functionally Loaded Subperiosteal Implants Evaluated on a Realistic Model Reproducing Severe Atrophic Jaws

**DOI:** 10.3390/mps8010008

**Published:** 2025-01-18

**Authors:** Gerardo Pellegrino, Maryia Karaban, Veronica Scalchi, Marco Urbani, Amerigo Giudice, Carlo Barausse, Pietro Felice

**Affiliations:** 1 Department of Biomedical and Neuromotor Sciences, University of Bologna, 40125 Bologna, Italy; gerardo.pellegrino2@unibo.it (G.P.);; 2 3D Product Specialist, Custom Made Medical Devices, BTK Dental, 36031 Dueville, Italy; 3 Department of Health Sciences of the ‘Magna Graecia’, University of Catanzaro, 88100 Catanzaro, Italy

**Keywords:** juxta-osseous implants, severe jaw atrophy, finite element analysis (FEA)

## Abstract

Implant-supported prosthetic rehabilitation for patients with severely atrophic jaws is challenging due to complex anatomical considerations and the limitations of conventional augmentation techniques. This study explores the potential of subperiosteal (juxta-osseous) implants as an alternative solution, using finite element analysis (FEA) to evaluate mechanical performance. Realistic jaw models, developed from radiographic data, are utilized to simulate various implant configurations and load scenarios. Results indicate that different screw placements, implant designs, and structural modifications can significantly influence stress distribution and biomechanical behavior. Upper and lower jaw models were assessed under multiple load conditions to determine optimal configurations. Findings suggest that strategic adjustments, such as adding posterior screws or altering implant connections, can enhance load distribution and reduce stress concentration, particularly in critical areas. Tensile loads in critical bone areas near cortical fixing screws exceeded 50 MPa under anterior loading, while configurations with larger load distributions reduced stress on both implant and bone. The study provides evidence-based insights into optimizing subperiosteal implant design to improve stability, longevity, and patient outcomes.

## 1. Introduction

It is widely recognized that implant-supported fixed prosthetic rehabilitation for patients with severely atrophic jaws poses significant challenges for clinicians [1]. Current surgical options for bone augmentation and subsequent standard-length implant placement include sinus lift, inlay/onlay bone grafting, guided bone regeneration (GBR) with resorbable or non-resorbable membranes, and distraction osteogenesis [2,3]. However, these methods typically require multiple surgeries, which can lead to post-operative morbidity, graft failure, prolonged treatment timelines, and significant costs [4,5,6].

As an alternative to the bone augmentation procedures, reduced-length or reduced-diameter implants can be considered. While short and ultrashort implants are viable options, they necessitate a minimum of 6 mm of bone height above the mandibular canal and at least 4 mm of bone height below the maxillary to host the shortest implants available [6,7]. Additionally, a minimum of 5 mm of crestal residual width is needed to place 3 mm diameter implants. In cases of extreme jaw atrophy, meeting these conditions may be impossible due to the proximity of critical anatomical structures, making the placement of short, ultrashort, and narrow implants challenging or even impossible [8].

Another potential solution is the use of tilted implants, which involves positioning standard implants in less resorbed areas of the jaw and angling them distally or mesially to achieve prosthetic rehabilitation of the adjacent site as well. However, tilted implants may be at risk of biomechanical failure, and the angulation is always dependent on the type of abutment used [9,10].

Zygomatic and pterygoid implants aim to circumvent the need for bone regeneration augmentation but present their own set of challenges. These procedures carry inherent risks of intra- and post-operative complications, are associated with high costs, and require the expertise of a highly experienced surgeon [11].

In addition to the latest advancements in biomedical technology, such as of computer-aided design (CAD)/computer-aided manufacturing (CAM) and personalized medicine, new trends in personalized medicine, including laser melting techniques, are emerging [12,13,14,15]. These innovations promise not only enhanced mechanical precision but also reduced surgical complexity, leading to potentially faster recovery times and improved patient outcomes.

In contrast to traditional methods, subperiosteal implants, also known as juxta-osseous implants, offer an alternative for implant-supported prosthetic rehabilitation. These implants are typically made of titanium and feature a three-dimensional design with a basal frame and wings that include holes for cortical bone fixation using screws [16]. Subperiosteal implants are placed directly on the jawbone and covered with periosteum, with transmucosal tunnel connections. The structure can be configured into separate right and left components or interconnected with an intraoral connector, depending on the residual anatomy of the jaw [17].

Dynamic mechanical loading significantly influences the stress distribution and biomechanical behavior of dental implants and increases stress magnitudes by 30–60% compared to static and quasi-static conditions, potentially leading to bone remodeling and implant fatigue [18]. However, stress concentrations in critical areas, such as the implant–bone interface, remain a challenge, especially under dynamic loading scenarios, and could contribute to issues like screw loosening and bone loss.

Despite several studies and the scientific literature, there are currently no standardized protocols defining the criteria for designing these implants to minimize stress on the structure and reduce impact on the patient’s bone [19,20,21,22,23]. The number and positioning of screws, among other factors, can influence the success of the treatment. To evaluate these parameters clinically, a significant number of patients would be needed for reliable results. In industrial settings, biomechanical feedback can help address this issue by assessing the design and positioning of screws. This approach involves biomechanical feedback related to the structural design and configuration of the implants [24,25,26].

Therefore, the aim of this study is to conduct mechanical and stress evaluations using finite element analysis (FEA) on anatomically accurate jaw models of patients. FEA is crucial for simulating and assessing mechanical performance, helping to optimize implant design for enhanced stability, longevity, and patient comfort [27,28]. To achieve these results, realistic models must be prepared to obtain biomechanical feedback, including cortical and medullary bone thickness and elastic modulus [29,30,31,32,33,34,35]. This approach not only facilitates theoretical predictions but also informs clinical decision-making by providing evidence-based insights into the biomechanical behavior of subperiosteal implants in real-world applications [36,37,38,39,40].

The study also aims to bridge the gap between technical advancements and patient-centered outcomes by demonstrating how optimized stress distribution can translate to reduced surgical complexity, improved recovery times, and enhanced long-term success rates. The object of the study is to evaluate differences in implant configuration, considering screw positioning and number, extension, design, and stress under various axial and non-axial loads, both in the anterior and posterior regions, while highlighting practical benefits for patients and clinicians alike. It will also assess the biomechanical response of realistic bone models.

## 2. Materials and Methods

A case involving extreme mandible and maxilla was selected, both presenting typical characteristics, thereby excluding extreme cases of bone atrophy [41]. The Digital Imaging and Communications in Medicine (DICOM) files, obtained from the radiographic examination, were segmented to isolate only the bone volume with the help of the RealGuide 5.2 software. Surface tessellation language (STL) of the portion of the skull below the eye sockets (upper part) and of the entire mandible (lower part) were obtained. The surfaces and volumes extrapolated from the radiographic examination were subjected to processing in order to repair small defects and to simplify the geometry. These operations were performed using Meshmixer 3.5 software. Through the same software it was also possible to recreate the cancellous bone volume where present, as the border between cortical and spongy bone is not always clear after the direct export of this volume starting from the radiographic examination. Cortical bone thickness measurements were therefore performed in various areas of the model, and the cancellous bone volume was obtained by offset with respect to the previously extracted bone surfaces.

Following the standard design process, the juxta-osseous implant was modeled onto the previously processed STL files. A preliminary draft of the prosthetic structure was also developed to position the abutments accurately and to identify the points of application for masticatory loads. Using reverse engineering Cyborg3D MeshToCAD software, parametric surfaces were reconstructed from the STL files, which are more compatible with current software. The files for the mandible, maxilla, and juxta-osseous implant were then converted to STEP format. Using SolidEdge 2022 software, additional details were added to the model, such as the cortical fixation screws. These screws were modeled in a simplified manner as excessive detail in the geometry would have led to excessively long computation times and overly complex results for the purpose of the analysis.

The next phase of the study focused on defining contact properties between different components of the system. Two types of contact were established:

- Glued contact: this type permanently bonds surfaces, preventing detachment or sliding.
- Frictional contact: allows surfaces to slide and detach from each other.

Specific surfaces within the geometric model were identified and paired using connectors, each assigned one of these contact properties:

- Between bone support and juxta support surfaces: frictional contact simulates realistic interaction.
- Between cortical bone and spongy bone surfaces: glued contact simulates their natural bond.
- Between screw threads and bone surfaces: simplified with glued contact due to screw geometry and assumed osseointegration.
- Between screw underhead and juxta seats: contact type property allows small displacements.
- Between juxta abutments and prosthesis: glued contact solidifies the implant-to-prosthesis connection.

Initial settings ensured zero penetration or gap, eliminating minor geometric imperfections. Materials were defined as isotropic and linear, with average bone characteristics adopted. Meshing employed tetragonal elements adaptable to varying geometries. For the characterization of the bone, average values were adopted since the properties vary depending on patient factors such as age, physiology, and pathologies of the patient.

Mesh creation proceeded using tetrahedral elements for all geometries of the model. The element sizes set were as follows: 1.5 mm for the general cortical bone, 1 mm for implant support areas, and 0.5 mm for cortical screw holes. For the spongy bone, element sizes were 1.5 mm for general areas and 1 mm for cortical screw holes. Juxta-osseous implants were meshed with 0.7 mm elements in general areas and 0.5 mm at cortical screw sites. Cortical screws were meshed with 0.5 mm elements. The prosthesis was meshed with 1.5 mm elements. These element sizes were chosen to ensure an accurate representation of the various geometries and areas of interest in the FEA model.

**Figure 1:**
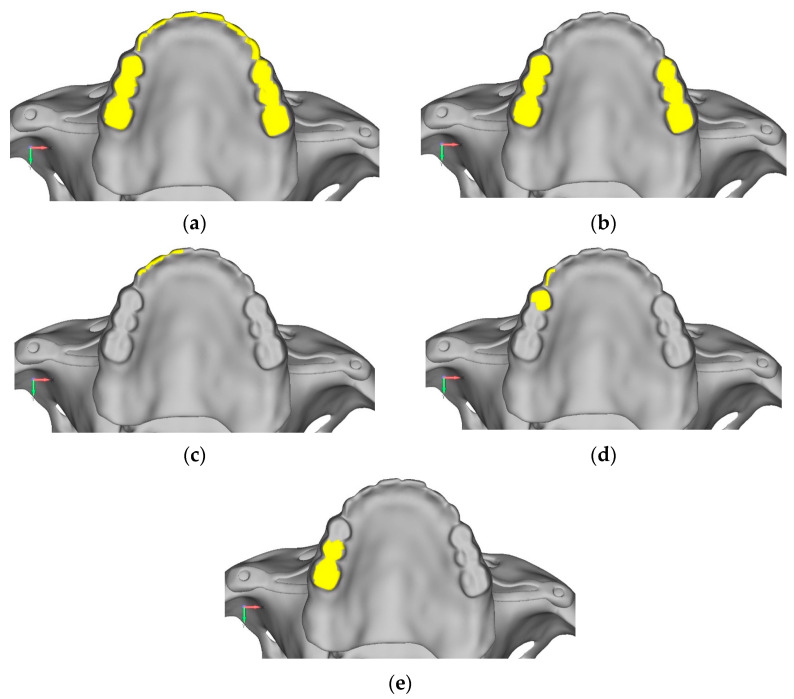
Different types of loads, indicated in yellow, were applied to the upper jaw. (**a**) Configuration 1: a uniform load is applied, distributed evenly across the entire jaw. (**b**) Configuration 2: a bilateral load is applied specifically in the molar region. (**c**) Configuration 3: an anterior unilateral load is applied to one side of the jaw. (**d**) Configuration 4: a unilateral load is applied in the premolar region. (**e**) Configuration 5: a unilateral load is applied in the molar region.

**Figure 2:**
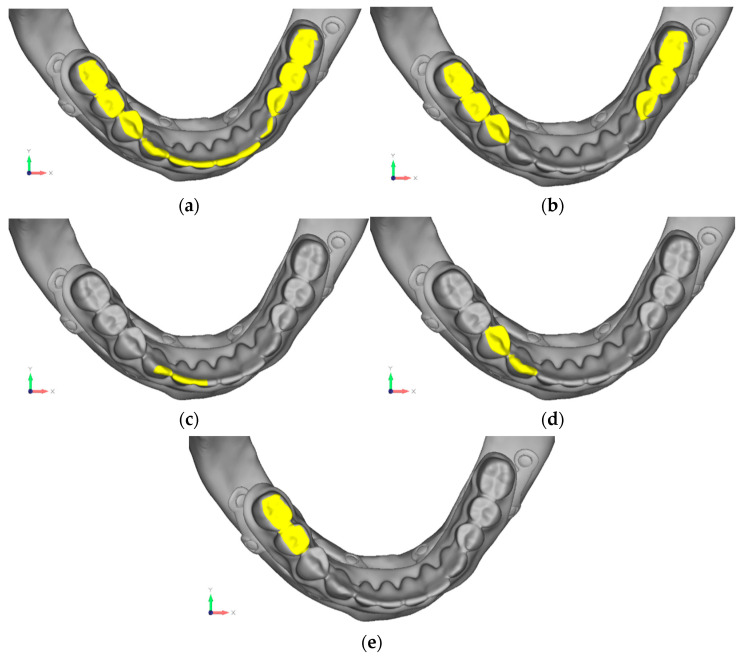
Different types of loads, indicated in yellow, were applied to the lower jaw. (**a**) Configuration 1: a uniform load is applied, distributed evenly across the entire jaw. (**b**) Configuration 2: a bilateral load is applied specifically in the molar region. (**c**) Configuration 3: an anterior unilateral load is applied to one side of the jaw. (**d**) Configuration 4: a unilateral load is applied in the premolar region. (**e**) Configuration 5: a unilateral load is applied in the molar region.

The term σmax allowable refers to the maximum stress that the material can withstand. In the case of titanium, this corresponds to the yield strength, while in the case of bone, it is a value derived from the literature and represents a safety load sufficient to prevent resorption effects. Loads were applied through node displacements in five configurations, each analyzed to assess structural responses under different load placements. The loads were applied by imposing displacements on specific nodes of the model. Specifically, the displacements were applied in 5 different configurations, each of which was analyzed individually to evaluate how the structure responded based on the point of load application (Figure 1a–e and Figure 2a–e).

All loads were static and vertically oriented, reflecting masticatory loads of 500 N. A stress limit of 50 MPa on bone was identified to prevent resorption effects (Table 1).

**Figure 3:**
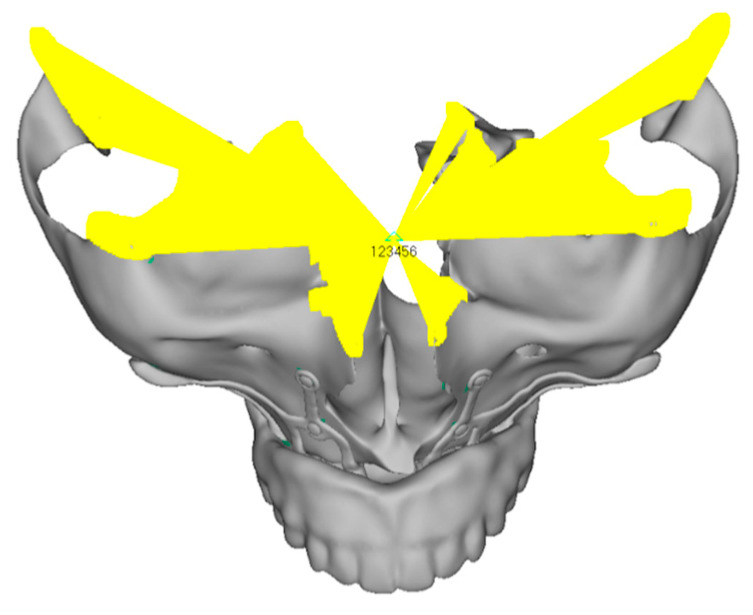
An image of the upper model, showing the “rigid” element in yellow and the central node with all six degrees of freedom locked (translations in x, y, z and rotations in x, y, z).

The upper model was constrained through the sectional surfaces of the cranial portion. The nodes on these sections were fixed (all degrees of freedom were locked). This constraint was applied using a “rigid” element, which connects the nodes adjacent to the surfaces to a central node. In this way, all the nodes linked to this element are fixed relative to the reference system (Figure 3).

**Figure 4:**
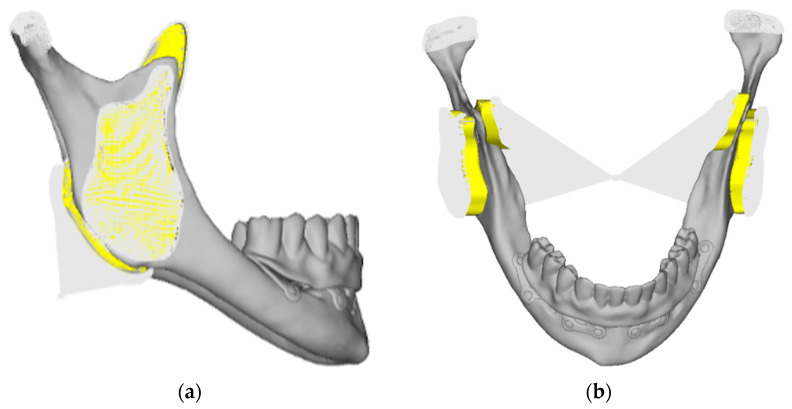
(**a**) An image of the lower model. The elastic pads were connected to a central node, which was constrained in all six degrees of freedom (translations in x, y, z and rotations in x, y, z). (**b**) The nodes corresponding to the condyles were constrained to two separate nodes (one for the right condyle and one for the left), which were further constrained only in translations (x, y, z). This setup represented a hinge corresponding to the mandibular joint.

A different approach was adopted for the mandible. The presence of muscles was simulated by constraining the mandible using elastic pads made of a material with a much lower elastic modulus than the materials in the model. These pads were then constrained to the reference system, creating a deformable junction between the model and the constraint. At the condyles, a hinge was created to allow rotation of the joint. This approach makes the stress distribution in the bone more realistic. In this case, “rigid” elements were also used to connect the surface constraints to single nodes (Figure 4a,b).

Thus, the process began by segmenting DICOM files to generate STL models of the skull and mandible, which were then repaired and simplified using Meshmixer. Cancellous bone was recreated, and cortical bone thickness was measured. The juxta-osseous implant was modeled on these processed files, with details added using Cyborg3D and SolidEdge CAD. Contact properties were defined, with glued and frictional connections applied. Isotropic material properties were used, and the model was meshed with specific element sizes. Loads of 500 N were applied, and a stress limit of 50 MPa was set to prevent bone resorption.

## 3. Results

As mentioned above, various load configurations were adopted. In the analysis of the first model (V0), the configuration that generated the highest stresses in the system was identified. All subsequent models were computed for all load configurations, but the optimization of the geometry was developed based on the most severe condition. The stresses are indicated in MPa.

**Figure 5:**
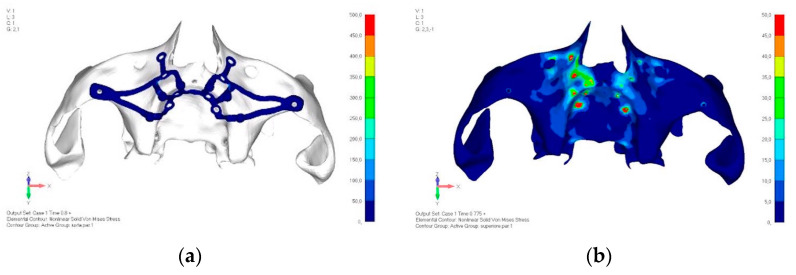
(**a**) Design of the subperiosteal implant in upper jaw model V0. (**b**) Model V0 with the most critical anterior right side load configuration 3.

**Figure 6:**
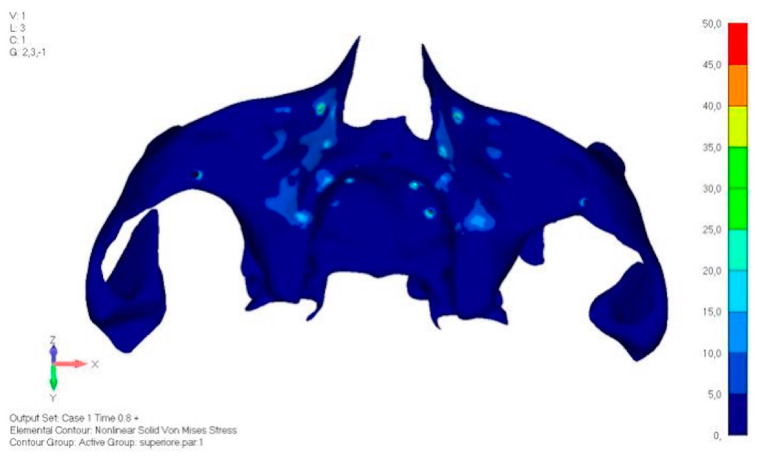
Model V0 with the least critical load configuration 1 distributed across the entire dentition.

**Figure 7:**
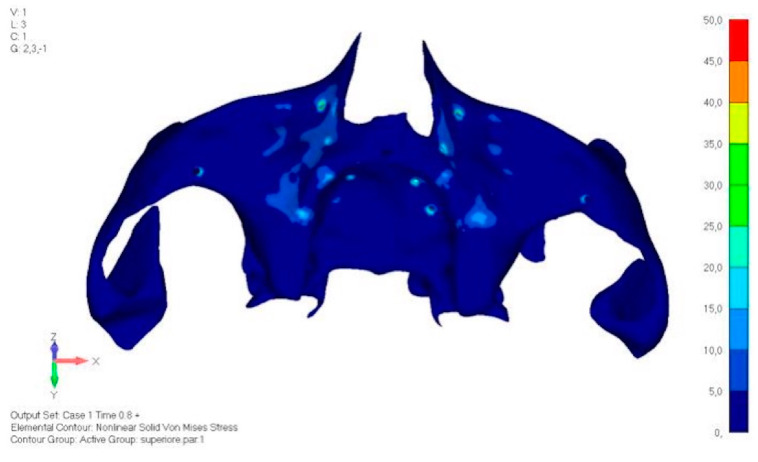
Model V0 with the least critical load configuration 2 distributed across the posterior teeth.

**Figure 8:**
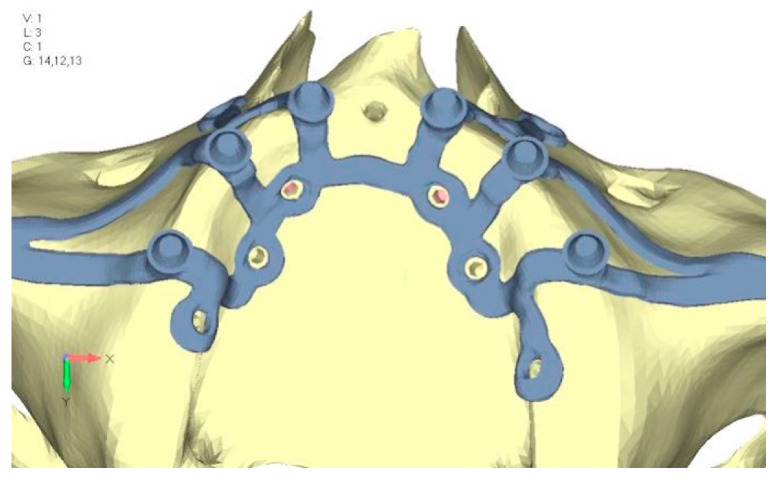
Design of the subperiosteal implant in upper jaw model V1 with added posterior screws, reducing stress on anterior parts and achieving more balanced distribution.

**Figure 9:**
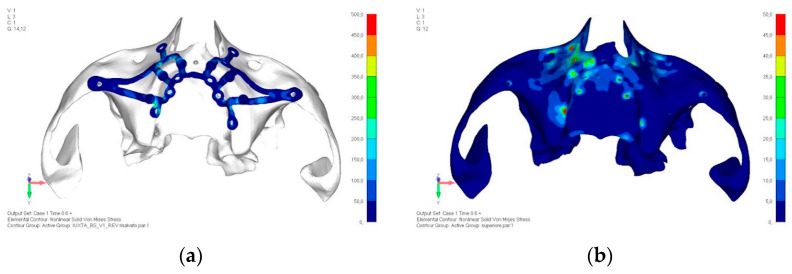
(**a**) Design of the subperiosteal implant in upper jaw model V1. (**b**) Upper jaw model V1 with added posterior screws, reducing stress on anterior parts and achieving more balanced distribution.

**Figure 10:**
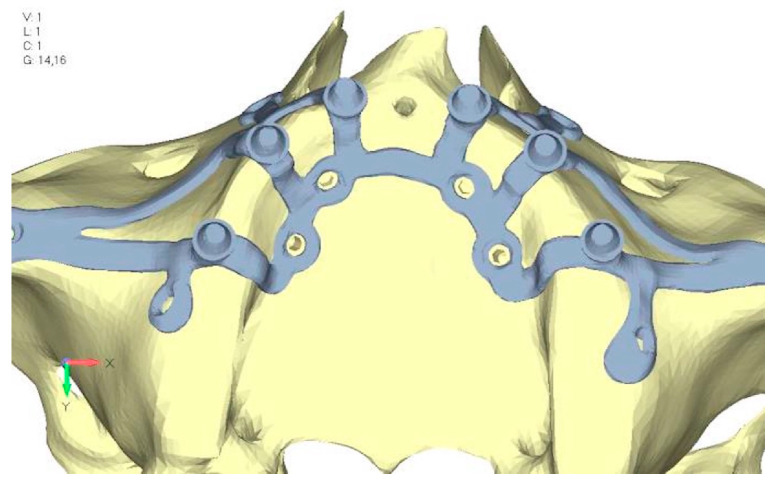
Design of the upper jaw model V2 structure using screws placed posteriorly in the vestibular direction rather than the palatal direction.

**Figure 11:**
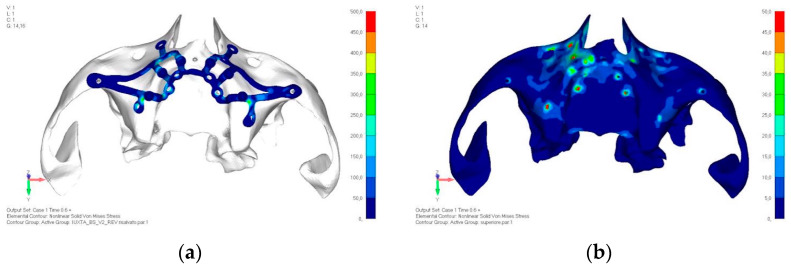
(**a**) Design of the subperiosteal implant in upper jaw model V2. (**b**) Loading distribution across upper jaw model V2, similar in behavior to model V1.

**Figure 12:**
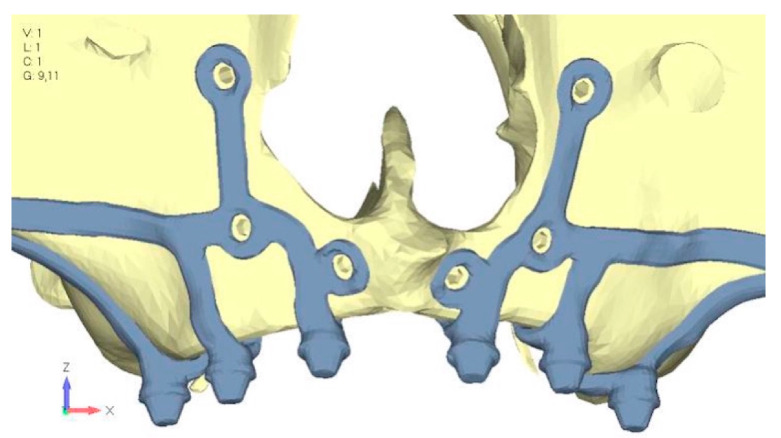
Design of upper jaw model V3 using screws placed in the vestibular direction rather than the palatal direction.

**Figure 13:**
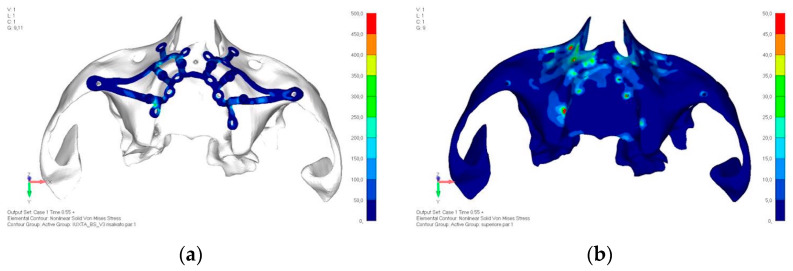
(**a**) Design of the subperiosteal implant in upper jaw model V3. (**b**) Upper jaw model V3 with optimized front section with additional screws, the stress on the frontal screws remained unchanged.

**Figure 14:**
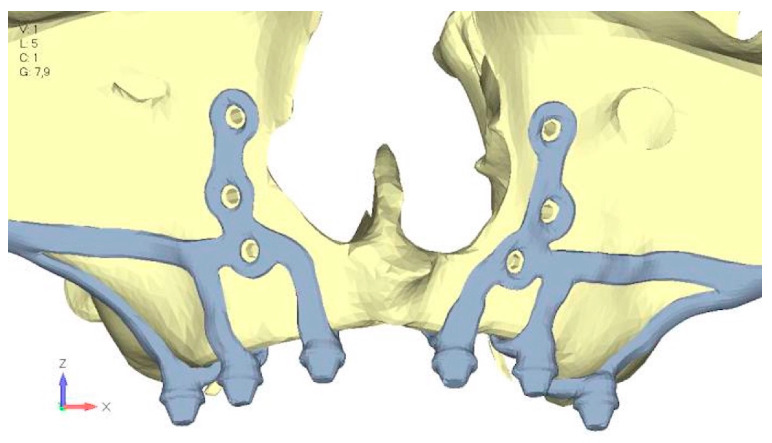
Design of upper jaw model V4 with the added screws relocated towards the frontal process, aligning them vertically with the other screws and ensuring that both arms of the first and second abutments connect to this screw.

**Figure 15:**
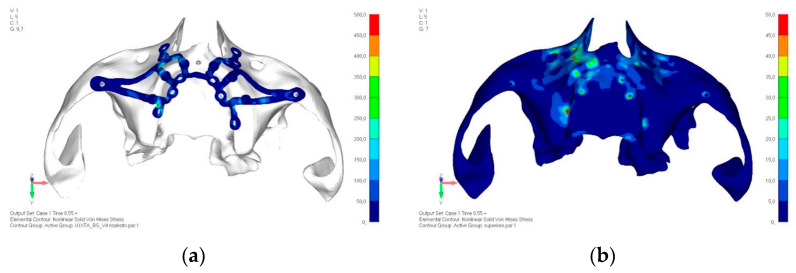
(**a**) Design of the subperiosteal implant in upper model V3. (**b**) Stress loading in upper model V4 exceeds 50 MPa in the vicinity of the screws. The crestal support shows stresses between 30 and 35 MPa.

**Figure 16:**
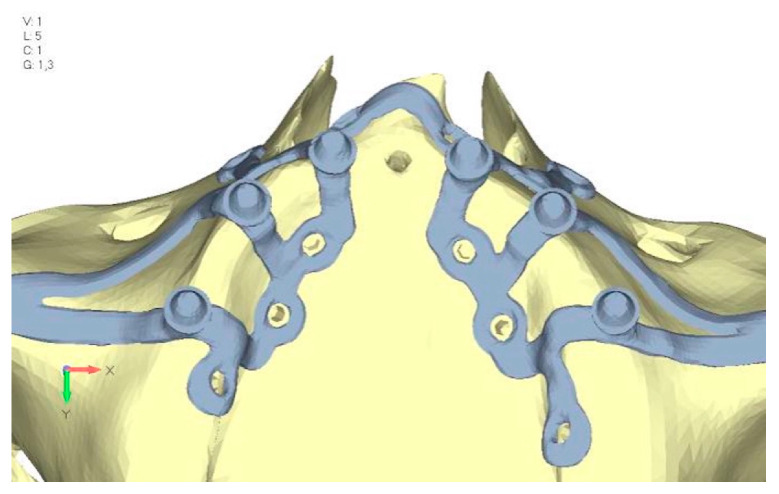
Design of model V5 with the connection between the two hemi-implants, affecting the structure. A frontal connecting bar was added while the palatal bar was removed.

**Figure 17:**
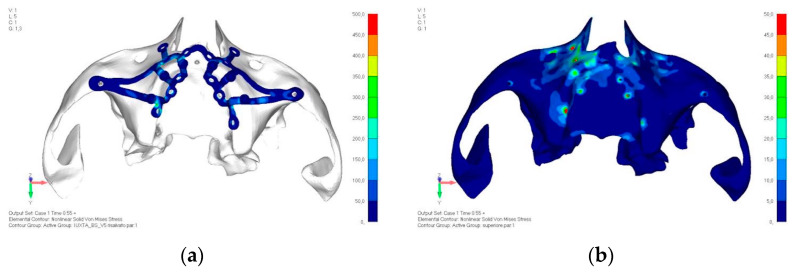
(**a**) Design of upper jaw model V5. (**b**) Stress loading in model V5 with the frontal connecting bar.

**Figure 18:**
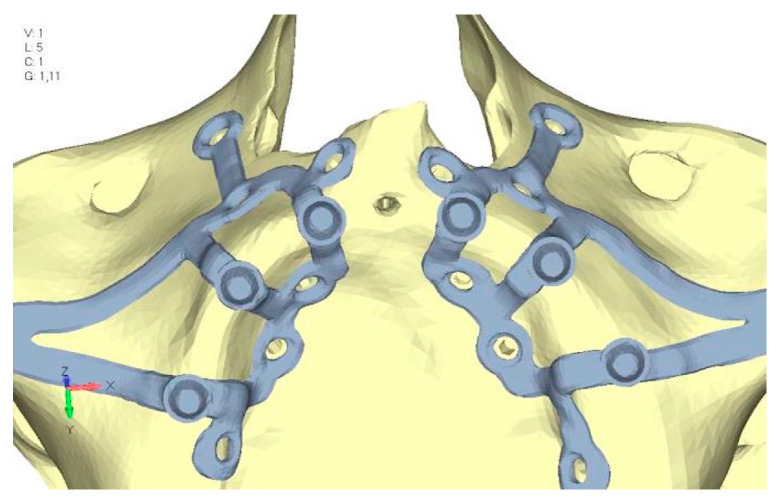
Design of model V6 divided into two hemi-arches without any connecting element.

**Figure 19:**
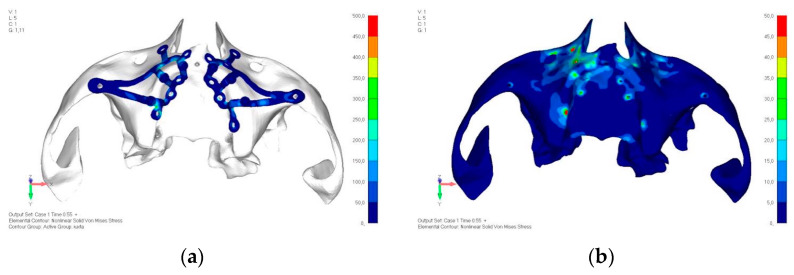
(**a**) Design of upper jaw model V5. (**b**) Stress loading in model V6 on the bone and implant remains the same as in cases with the connection.

Upper jaw models:

- Model V0. From the analysis of Model V0, it emerged that the most critical situation is related to load configuration 3 (Figure 5a,b), which represents a load applied to the anterior right side. The least critical situations are load configurations 1 and 2 (Figure 6 and Figure 7), corresponding to a load distributed across the entire dentition and a load distributed only on the posterior teeth, respectively. Regarding the stress values observed, no critical issues were identified with the juxta-osseous implant. In load configuration 3, the stresses are below the breaking limits of titanium laser melting: peak stresses of 500 MPa are reached only in very localized areas of the implant.
- Model V1. Added posterior screws, reducing stress on anterior parts and achieving more balanced distribution. The addition of the posterior screw has certainly alleviated the load on the palatal screw, which was excessively stressed in the previous model (Figure 8). The screw now experiencing the most stress is the posterior screw: compared to the previous case, only part of the hole shows a stress exceeding 50 MPa, and the area affected by this stress is therefore much more contained (Figure 9a,b).
- Model V2. This model serves as an alternative to model V1, as it aims to stabilize the structure posteriorly using screws placed in the vestibular direction rather than the palatal direction (Figure 10). The model displayed similar behavior to V1, leading to the decision to proceed with V1 for further development (Figure 11a,b).
- Model V3. Based on the findings from model 1, attention was shifted to the anterior section to optimize the anchors in that area. Two additional screws were placed anterior to the nasal spine to reduce the load on the frontal screws (Figure 12). The analysis revealed minimal changes; the stress on the frontal screws remains the same, while the pressure on the anterior crestal support has decreased to below 35–40 Mpa (Figure 13a,b).
- Model V4. The previously added screw was relocated towards the frontal process, aligning it vertically with the other screws and ensuring that both arms of the first and second abutments connect to this screw (Figure 14). This solution proved to be more effective than V3; the addition of the screw reduces the stress on the other screws and on the support. The area where stress exceeds 50 MPa in the vicinity of the screws is now more contained, and the crestal support shows stresses between 30 and 35 MPa, which are absolutely acceptable (Figure 15a,b).
- Model V5. This model determined whether the connection between the two hemi-implants affects the behavior and stability of the implant. Specifically, in this model, a frontal connecting bar was added while the palatal bar was removed (Figure 16 and Figure 17a,b).
- Model V6. This model analyzes an implant divided into two hemi-arches without any connecting element. As can be easily observed, the presence or absence of an element joining the two halves of the implant has no effect on the stress state of the model. In all previously analyzed models, the bar connecting the two hemi-arches of the implant shows no stress (Figure 18). Removing this bar in model 6 does not alter the results in any way; the stress state of the bone and implant remains the same as in cases with the connection (Figure 19a,b).

This effect is clearly due to the presence of the prosthesis, which is not visualized in the model but is included in the numerical calculations. The prosthesis helps to stiffen the system without the need for a connecting element at the implant level. As can be easily observed, the presence or absence of an element joining the two halves of the implant has no influence on the stress state of the model. In all previously analyzed models, the bar connecting the two hemi-arches of the implant shows no stress. Removing this bar in model 6 does not alter the results in any way; the stress state of the bone and implant remains the same as in cases with the connection.

Lower jaw models:

The same approach as the upper jaw was adopted; the model was analyzed in different load configurations to identify the most onerous situation.

**Figure 20:**
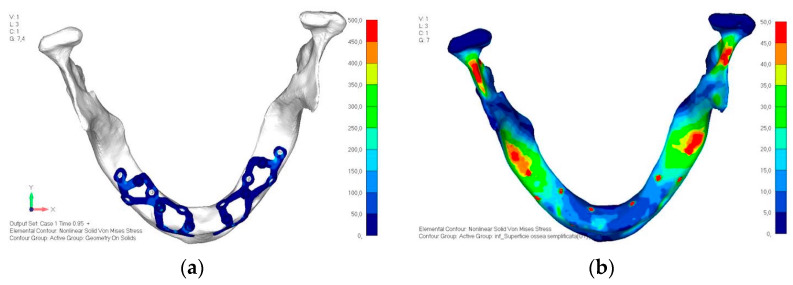
(**a**) Design of the subperiosteal implant in lower jaw model V0. (**b**) The most significant load on lower jaw model V0, corresponding to chewing in the anterior right sector.

**Figure 21:**
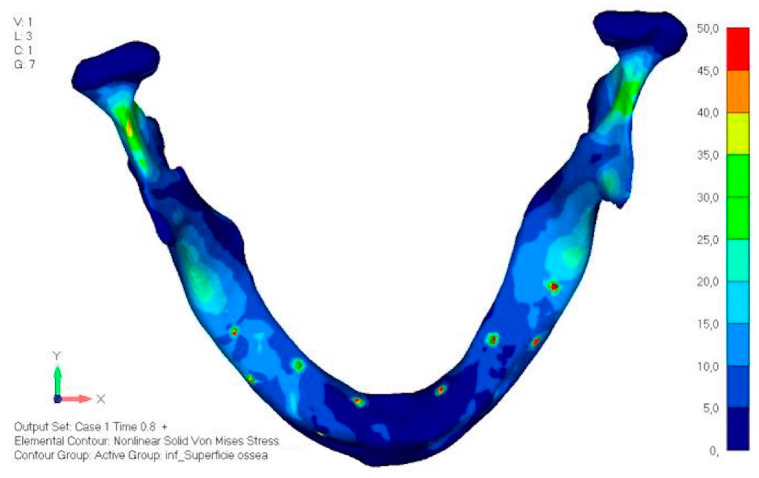
Load configuration 1 distributed over a larger area of lower jaw model V0.

**Figure 22:**
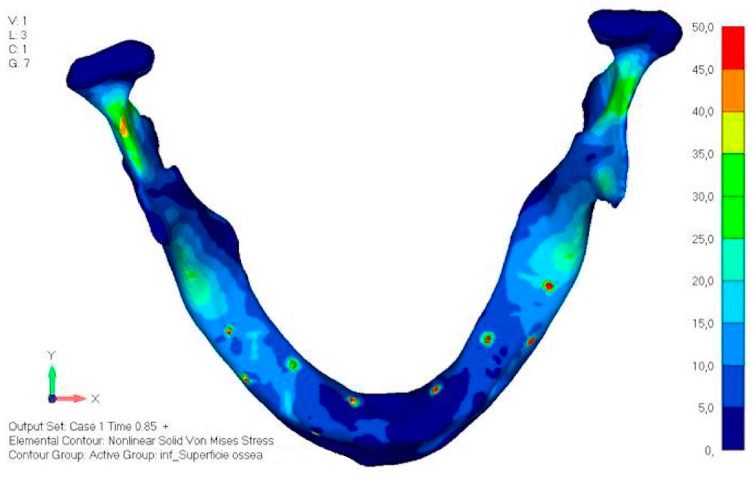
Load configuration 2 distributed over a larger area of lower jaw model V0.

**Figure 23:**
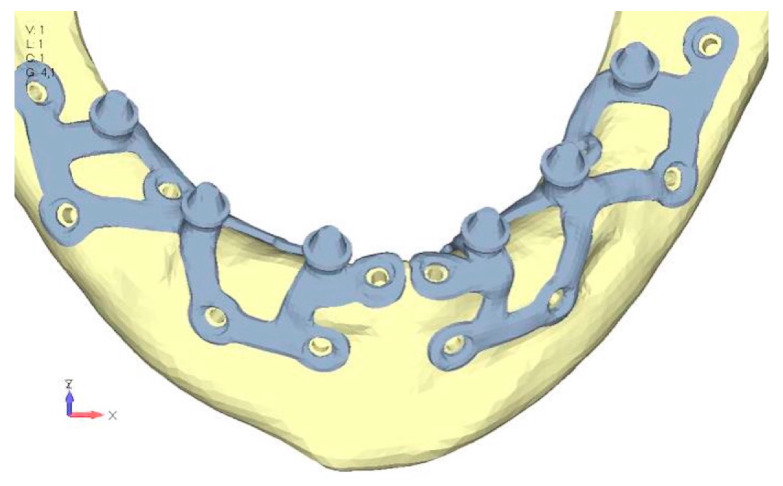
Design of model V1 with two anterior appendages added in a crestal position.

**Figure 24:**
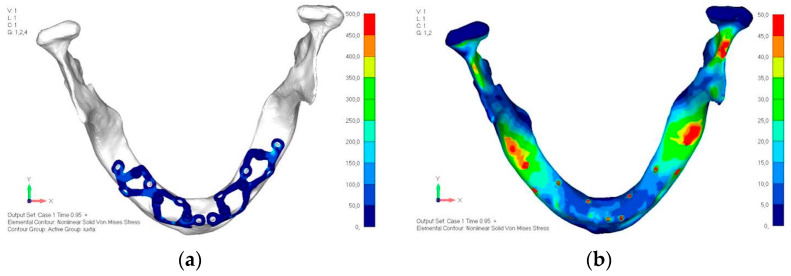
(**a**) Design of the subperiosteal implant in lower jaw model V1. (**b**) Stress loading in model V1 and implant, with no improvement in the configuration observed.

**Figure 25:**
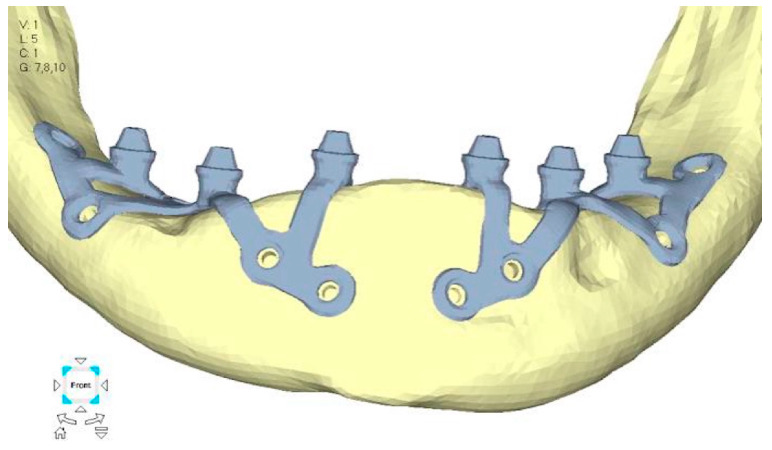
Design of model V2 with extended anterior vestibular arms that connect to the first abutment.

**Figure 26:**
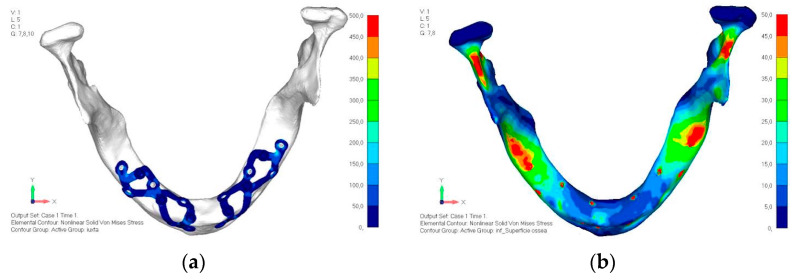
(**a**) Design of the subperiosteal implant in lower jaw model V2. (**b**) The stresses near the holes in model V2 are similar to those observed in model V1.

**Figure 27:**
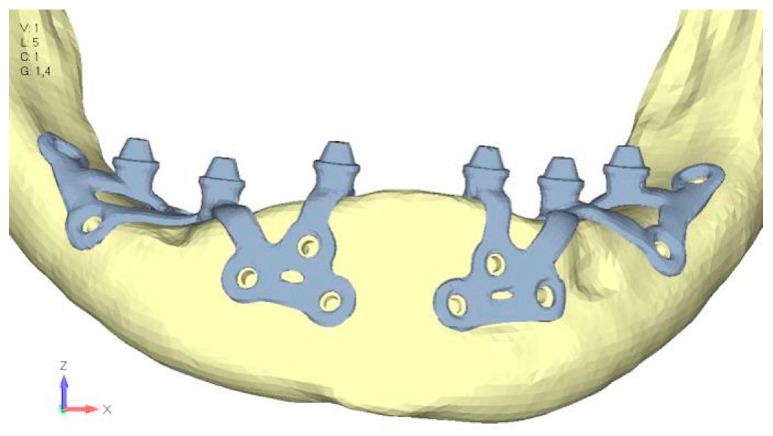
Model V3 design with an additional screw, distributing the load of the anterior abutment across three screws instead of two.

**Figure 28:**
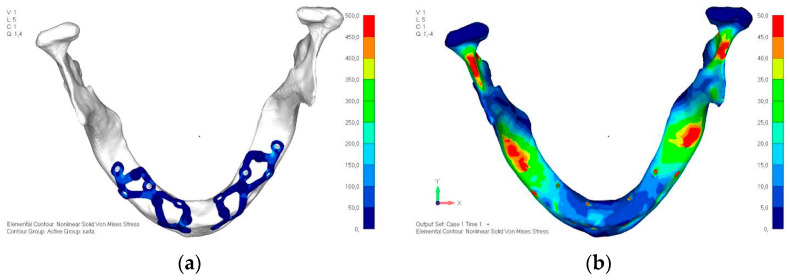
(**a**) Design of the subperiosteal implant in lower jaw model V3. (**b**) Decreased stress loading on the material of model V3.

**Figure 29:**
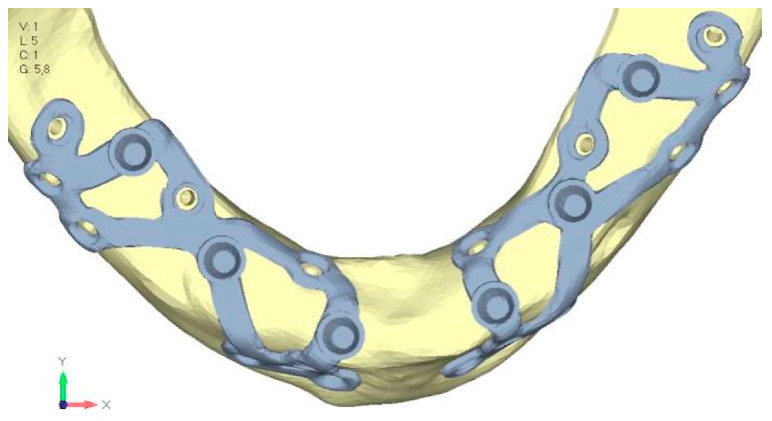
Model V4, design 3, adds a screw in the posterior sector, positioned in the vestibular direction to model V3.

**Figure 30:**
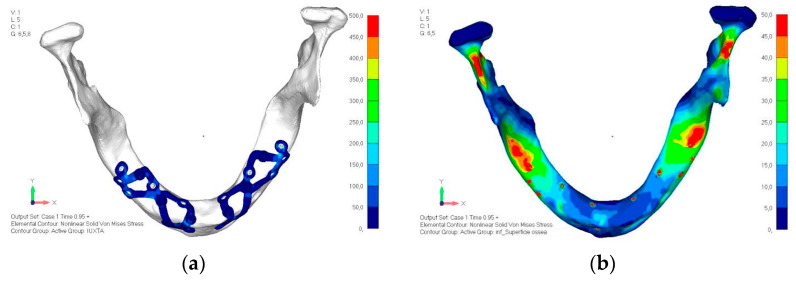
(**a**) Design of the subperiosteal implant in lower jaw model V4. (**b**) The posterior alveolar area of model V4, particularly around the more posterior screws, remains notably stressed.

**Figure 31:**
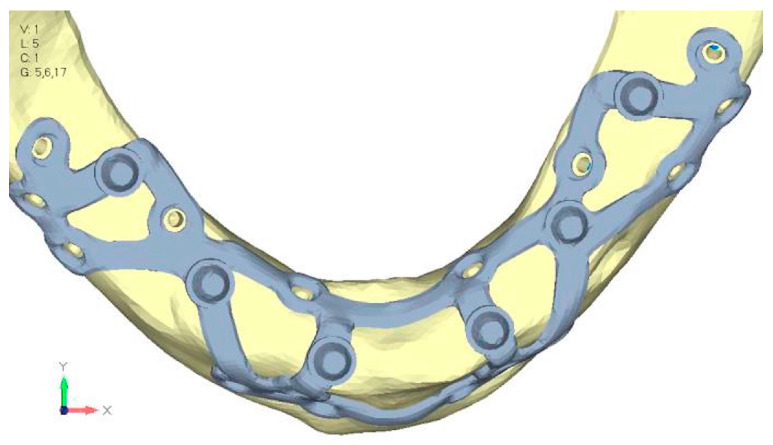
Model V5 design with the same geometry as version 4, with the addition of two connecting bars, one on the lingual side and one on the vestibular side.

**Figure 32:**
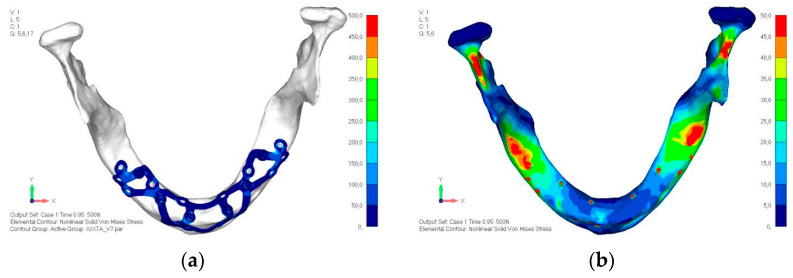
(**a**) Design of the subperiosteal implant in lower jaw model V5. (**b**) Stress loading in model V5 on the two connecting bars exhibits stresses close to 0, indicating that no force is transmitted through them.

- Model V0. This model represents the initial analysis performed on the lower arch. The implant consists of two completely separate hemi-arches. The situation observed in the lower model is very similar to that found in the upper model. The most significant load is load 3 (Figure 20a,b), corresponding to chewing in the anterior right sector. Loads distributed over larger areas, such as configurations 1 and 2, result in less stress on both the implant and the bone (Figure 21 and Figure 22).
- Even in the lower model, the stresses observed in the peri-implant bone are always acceptable and significantly lower compared to those found in the upper implant. In the worst case, peak stresses reached 250 MPa, which ensures an adequate safety margin. From the bone perspective, in load configuration 3, it is noted that stresses exceed 50 MPa even in areas distant from the implant, such as near the condyles and in the posterior alveolar process.
- Model V1. In this version of the implant, two anterior appendages have been added in a crestal position with the aim of better distributing the load in that area (Figure 23). The examined configuration does not result in improvements. Additionally, from a practical standpoint, it is unfeasible because the presence of the crestal screws would create an obstacle in managing the soft tissues, increasing the risk of dehiscence and exposure of the implant (Figure 24a,b).
- Model V2. The implant has been modified anteriorly by extending the anterior vestibular arms that connect to the first abutment (Figure 25). This change aims to achieve greater flexibility of the implant in that area, promoting the transmission of masticatory load to the bone through support rather than through the screws. The modification did not reveal significant changes in the stress state. The stresses near the holes are similar to those observed in model 1 (Figure 26a,b).
- Model V3. To reduce the load on the front screws, it was decided to add an additional screw, distributing the load of the anterior abutment across three screws instead of two (Figure 27). The addition of the anterior screw has certainly improved the distribution of stresses, as the volume of material experiencing stresses greater than 50 MPa near the screws has decreased (Figure 28a,b).
- Model V4. This version was derived from version 3 by adding a screw in the posterior sector, positioned in the vestibular direction (Figure 29). Again, the addition of an anchoring screw has allowed for more effective distribution of the stresses. The posterior alveolar area, particularly around the more posterior screws, remains notably stressed (Figure 30a,b). However, this phenomenon is attributed to the geometry and configuration of the bone rather than the presence of a cortical screw.
- Model V5. This version of the implant retains the same geometry as version 4, with the addition of two connecting bars, one on the lingual side and one on the vestibular side (Figure 31). The purpose of this analysis is to identify the differences between a monolithic implant and an implant divided into two hemi-arches. The results are quite similar to those observed in the upper model: the presence of a connection between the two halves of the implant does not contribute to its stability. It is immediately noticeable that the two connecting bars exhibit stresses close to 0, indicating that no force is transmitted through them (Figure 32a,b). Once again, a significant contribution is provided by the prosthesis, which stiffens the structure through the abutments.

## 4. Discussion

The present study aimed to simulate on a realistic anatomical model the design of juxta-osseous implants in order to evaluate the biomechanical stress on the implant and the bone. Cases concerning the mandible and the maxilla were selected for the study, presenting typical characteristics of severe bone atrophy [41], for which designing juxta-osseus implants for the future prosthetic rehabilitation could be a valid treatment option. A similar study was conducted by De Moore (2022), analyzing even more extreme cases of bone atrophy, and the design of subperiosteal implants was found to be completely safe and considered a great solution for implant-supported fixed rehabilitation [42].

This study focused on various load simulations and the design of juxta-osseous implants. The structural tension values were extrapolated from the literature, representing an average load [43]. The recent study of Ayhan (2023) showed that 1 mm thick implants show more displacement than 1.5 mm thick ones, even though the differences between the groups are negligible [44]. However, the analyses of the present study have highlighted that the metal structure of the subperiosteal implant effectively withstands masticatory loads, with no particular issues in terms of stresses. Therefore, the thicknesses of the metal structure adopted for the design (0.7 mm in the general area and 0.5 mm in others) were deemed suitable for the study’s purpose.

In studying different types of loading, Antiparmak (2023) revealed that the cortical bone experienced the highest minimum principal stress values under posterior oblique loading forces [45]. Meanwhile, Zielinski’s research (2023) highlighted that while vertical loads at 90 degrees induced minimal strain and stress, non-axial forces significantly escalated stress levels in multi-unit configurations, reaching nearly 500 MPa [46]. The present study focused exclusively on vertical static forces, which we acknowledge may limit its scope and represent a less comprehensive scenario compared to real-world conditions where oblique forces are prevalent. Further investigation into the effects of oblique forces is warranted to provide a more comprehensive understanding of the topic. The finite element mesh sizes used in this study were chosen based on prior experience to balance computational efficiency and accuracy. However, the absence of a formal mesh sensitivity analysis is acknowledged as a limitation.

The results of the present study indicated that the most significant load occurred during the anterior load, aligning these data with recently published studies [47,48,49,50]. Larger load distributions, as seen in configurations 1 and 2, resulted in reduced stresses on both the implant and bone. Critical areas in the bone, especially near cortical fixing screws, experienced tensile loads exceeding 50 MPa. The constant load applied across all dentition areas contributed to the stress distribution observed. It is preferable to have a lower masticatory force on the anterior sectors compared to the posterior ones.

The most stressed screw sites were the posterior and anterior vestibular ones. The presence of crestal screws was minimized to avoid clinical issues related to soft tissue management and reduce the risk of dehiscence and implant exposure. It is important to note that in Ayhan’s study, there is a specific emphasis on screw use being a more critical factor affecting bone stress than implant design. This underscores the importance of carefully evaluating screw positioning during surgical procedures [44].

The fixation of the implants in the anterior and posterior sectors was crucial: in the posterior area, the last screw was retracted as much as possible to increase the force generated by the screw. In the anterior sector, having a sufficient number of screws in the buccal direction is essential to support the shear loads. In the upper case, three screws were arranged, aligning them approximately vertically and laterally to the nasal cavity. In the lower model, the adequate number of screws was found to be three, which support the first and second abutments [46]. The study further explored the implications for patient outcomes, emphasizing that uniform stress distribution and lower anterior masticatory forces can enhance the durability and success of the implant system. Long-term clinical implications include the importance of optimizing prosthesis material and design to ensure stability and functionality. Future research will address oblique loading scenarios and patient-centric outcomes, such as improved comfort, long-term durability, and functional efficiency, to provide a more comprehensive understanding of juxta-osseous implant systems. The absence of a mesh sensitivity analysis is acknowledged as a limitation of this study. Future research should incorporate a comprehensive mesh sensitivity analysis to ensure the robustness, accuracy, and reliability of the findings, particularly in the context of clinical applications.

Although Ayhan’s (2023) study revealed that dual implants demonstrated lower von Mises stress within the implant structure compared to mono implants, mono implants exerted less force on the bone, leading to a more uniform load distribution across the upper jaw and, consequently, lower residual stresses in the bone [44]. There is no difference in the present study in bone tension and the structure’s tension between one-piece and two-piece implants. This effect could be due to the presence of the prosthesis, which helps to stiffen the system without requiring a connecting element at the implant level. It is worth noting that the material constituting the prosthesis in these models is dental engineering resin; if the prosthesis were made of a metallic material, it could further stiffen the implant–prosthesis system.

## 5. Conclusions

The present study highlighted the biomechanical characteristics of juxta-osseous implant under masticatory load. For both the upper and lower models, the heaviest load configuration was found to be the one with the load applied at the front. The analyses indicated that the metal structure of the juxta-osseous implant effectively withstands masticatory loads, with no critical issues regarding tension being identified. However, the proper fixation of the implants was crucial. No significant differences were observed between a one-piece implant and an implant divided into two parts in either model. The role of the prosthesis, which reinforces and stiffens the structure, was found to be essential. Nevertheless, to validate the results of this study, clinical trials are required.

## Figures and Tables

**Table 1:** The characteristics of the material and the mesh adopted from the literature.

Type of Material	Elastic Module E (MPa)	Poisson Coefficient	σmax Maximum Allowable (Mpa)
Cortical bone	13,700	0.3	50
Trabecular bone	1370	0.3	-
Titanium Gr5 (load model)	101,000	0.34	950
Titanium Gr5 (bar)	101,000	0.34	970
Resin for prosthetics	3000	0.3	-
Muscle simulators	25	0.4	-

## Data Availability

The original contributions presented in this study are included in the article. Further inquiries can be directed to the corresponding author.
